# The geranium genus: A comprehensive study on ethnomedicinal uses, phytochemical compounds, and pharmacological importance

**DOI:** 10.1016/j.sjbs.2024.103940

**Published:** 2024-01-28

**Authors:** Bader Alshehri

**Affiliations:** Department of Medical Laboratory Sciences, College of Applied Medical Sciences, Majmaah University, Almajmaah 11952, Saudi Arabia

**Keywords:** *Geranium*, Ethnobotany, Phytochemistry, Databases and drug discovery

## Abstract

*The geranium* genus consists of about 400 species, which have been utilized for a long time in ancient medical practices throughout the world. As a result, herbal medications based on species are commonly utilized to treat a range of illnesses. This investigation aims to provide an extensive assessment of the literature on the phytochemistry, ethnomedicinal and pharmacological importance of the genus *Geranium*. Data were collected through systemic computer searches among the most reputable scientific databases, Web of Science, Google Scholar, and Scopus. Occasionally, information published as peer-reviewed literature was added to data from sources that these databases do not include. This review includes all published works through the end of 2022. The assessment of the biological characteristics of medicinal plant species in the genus *Geranium* has received a great deal of attention, primarily in the last 20 years, in tandem with the growing interest in herbal remedies in general. The detailed and systematic comparative analysis presented here provides valuable information on the current *Geranium* species. It paves the way for other beneficial species of *Geranium* to be studied in the fields of ethnobotany, phytochemistry, and new drug discovery.

## Graphical abstract

**Figure:**
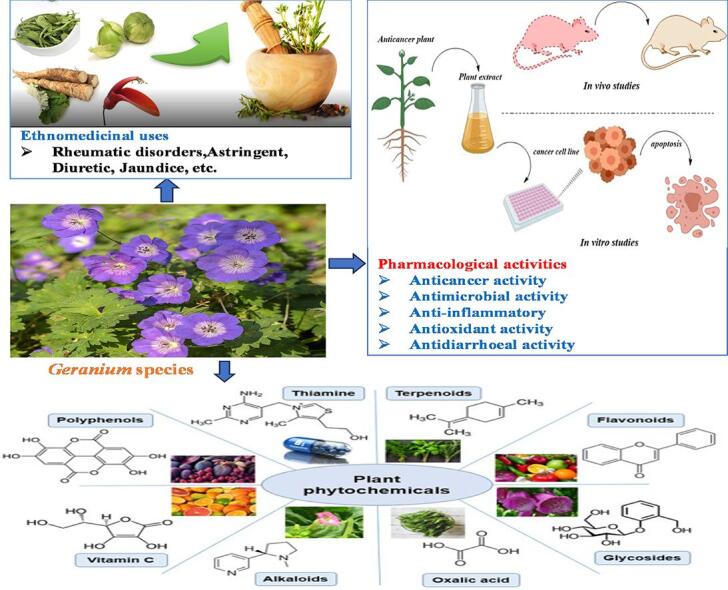

## 1 Introduction

The advancement of human civilization has coincided with the usage of plants as herbal remedies to cure a wide range of diseases (Choudhury et al., 2023, Lone et al., 2023). The World Health Organization estimates that, at the end of the 20th century, about 80 % of the world's population received their primary healthcare from traditional medicines, which mainly used plant extracts or their active ingredients (Tahir et al., 2023, Mir et al., 2022). Synthetic medications have greatly improved human health and are essential tools in the battle against a variety of diseases (Qayoom et al., 2023, Jamal, 2023). However, overuse of synthetic medications has been shown in recent years to have harmful consequences on human health (Krishnan et al., 2023, Sofi et al., 2023). Scientists are working to create new medications with fewer adverse effects. Since traditional medical systems are more natural, favourable to the environment, and free of side effects, they are gaining high popularity (Jan et al., Khan et al., 2021). Therefore, people still prefer plant-based natural cures over synthetic pharmaceuticals, even with all of the benefits that modern synthetic medications offer. Because they contain many vital phytoconstituents in distinct plant parts, most medicinal plants are unique in their ability to treat and cure a variety of human health issues (Anand, Jacobo-Herrera, Altemimi, & Lakhssassi, 2019). Medicinal plants include a variety of bioactive compounds with pharmacological effects, including anticancer, antibacterial, anti-inflammatory, and antioxidant properties (Bourais et al., 2023). The primary goal of this work is to provide a complete overview as possible of the scientific contributions supporting the traditional medicine and medical herbalism applications of plants belonging to the genus *Geranium*, which have particular biological activities.

The *Geranium* genus consists of about 400 species, most of which are distributed in the Northern Hemisphere, primarily in temperate climates (Graça, Ferreira, & Santos, 2020). Some species are distributed in tropical regions but in the montane environment at high altitudes. However, species also grow naturally in Australia, New Guinea, South and North Africa, and islands in the Pacific and Atlantic Oceans. Many species are cultivated from North America to Eurasia (Graça et al., 2020). In warmer climates, a large number of plants are annual, passing through the hottest season as seeds, or if perennial habit, they may have a tuber and disappear in summer. Most native species found in Alpine regions develop slowly. The perennial species, some of which are well over 1 m tall, are typically present in grasslands or at the edges of woods. There are 27 *Geranium* species in India, with maximum diversity in tropical, hilly regions, and temperate Himalayas, including the Deccan peninsula, north-eastern, and Western Ghats region. Kashmir Himalaya is home to one endemic species, *G. clarke* Yeo (Wagh et al., 2015). Since ancient times, the species of *Geranium* have been utilized in numerous conventional health systems across the globe, such as Indian Ayurveda, Traditional Chinese medicine, and various indigenous medical practices, by means of herbal formulations, (Bhat et al., 2022, Graça et al., 2020). Accordingly, it has been claimed that many plants in this genus are utilised to cure a variety of illnesses. *G. robertianum* has been used for the treatment of haemorrhage, diarrhoea, mouthwash, wounds, gall stones, and burns (Renda, Celik, Korkmaz, Karaoglu, & Yayli, 2016). *G. ruizii* have been used for the treatment of diabetes, inflammation, and chronic diarrhea (Ikeda et al., 2014). The species of the *Geranium* genus have significant pharmacological activities such as anticancer, antioxidant, anti-inflammatory, and antimicrobial properties (Bhat et al., 2022, Graça et al., 2020).

Systematic computer searches of large, well-known scientific databases, including “Web of Science” and “Scopus,” yielded relevant information. Periodically, data published as peer-reviewed literature that was sourced from primary sources not included in these databases was also incorporated. Information from primary or secondary sources that did not meet these two criteria was not taken into consideration for this review. To expand the knowledge base on the biological activities of the *Geranium* genus, this review also included species whose bioactivity is documented based on *in-vitro* or *in-vivo* research but were not reported to be employed in traditional medicine or herbalism practise. This review includes works that were released up until the end of 2022. The scientific names of plants were verified using the internet database “The Plant List” (Hidayat et al., 2023). This assisted in locating misspellings and the usage of synonyms for various species. If a species' botanical name was unclear or imprecise, it was eliminated. Very few scientific reports regarding the chemical profiling and pharmacological activities of extracts derived from *Geranium* species. Therefore, we must have up-to-date knowledge of this genus and its ethnomedicinal, phytochemical, and pharmacological uses, which will enable us to design future studies of this genus.

## 2 Botanical description of the *Geranium* genus

There are over 840 species in the Geraniaceae family (Kalwij, 2012). The plants of the genus *Geranium* are mainly annual or perennial shrubs or herbs globally distributed, primarily in subtropical and temperate climates (Fiz et al., 2008, Graça et al., 2020). This family’s species are categorized into six genera: *Geranium, Pelargonium, California, Monsonia, Hypseocharis,* and *Erodium*. The species are biennial, annual, herbaceous, or perennial, and some plants have woody bases; while some contain tubers. Petiolate leaves with toothed or lobed divisions that have stipules are usually aglandular or glandular-hairy and palmately divided. The lower leaves may be alternate; however, the stem leaves are typically opposite. The *Geranium* flowers are usually purplish, pink, or bluish-pink and are borne as solitary or pairs or in smaller umbels encircled by bracteoles. With five hairy sepals and a mucronate tip, the flowers are radially symmetrical. The size of sepals usually increases as the fruit matures. The nectary is near the base of the five equal petals, which can be clawed and occasionally have notches at the tips. Ten stamens are present in polyandrous flowers, grouped in two whorls, with the outer whorl’s anthers dehiscing before the inner whorl’s. In order to prevent self-pollination, the gynoecium has a style that is divided into 5 stigmas that mature after the dehiscence of anthers. After fertilization, the 5 mericarps each consist of only a single seed (Graça et al., 2020).

## 3 Phytochemistry of the genus *Geranium*

**Fig. 1:**
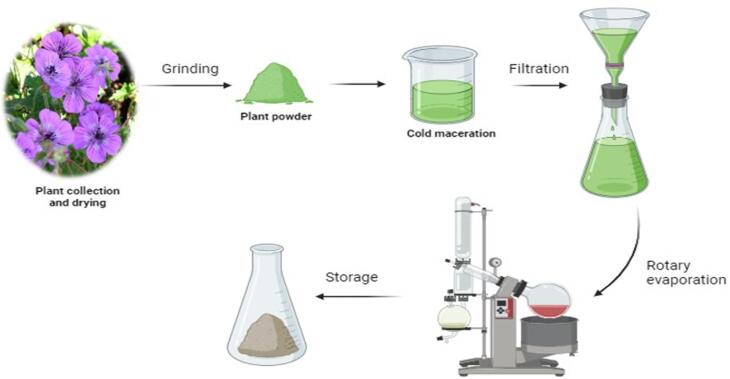
Extraction of plant material by cold maceration method using various solvents. The solvent used in extraction usually depends on the active constituent, e.g. alkaloids and glycosides, by using alcohol and water in different proportions.

**Fig. 2:**
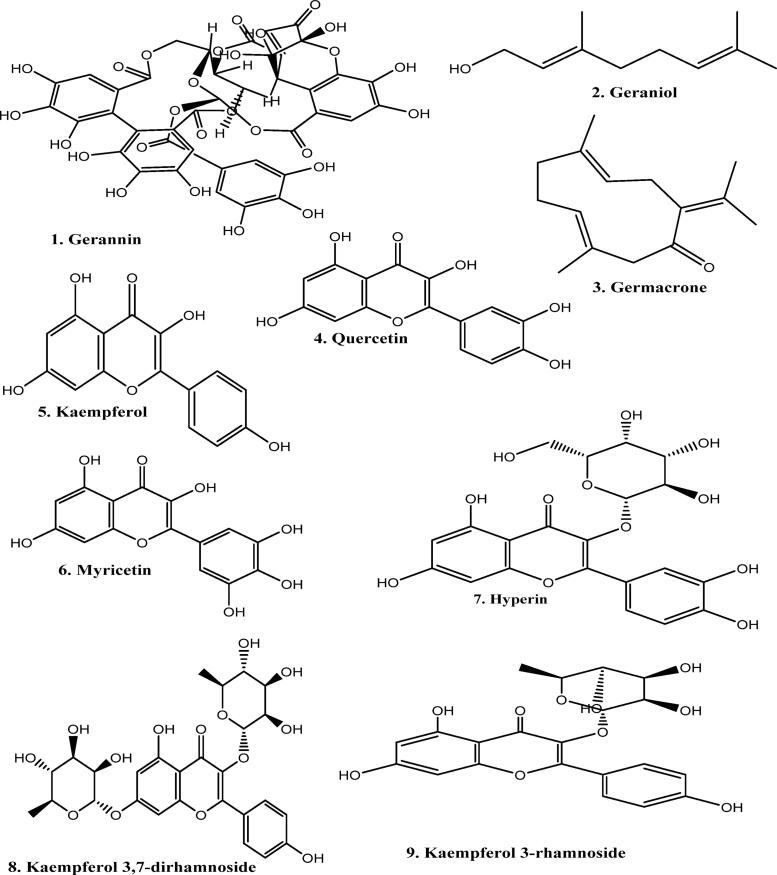

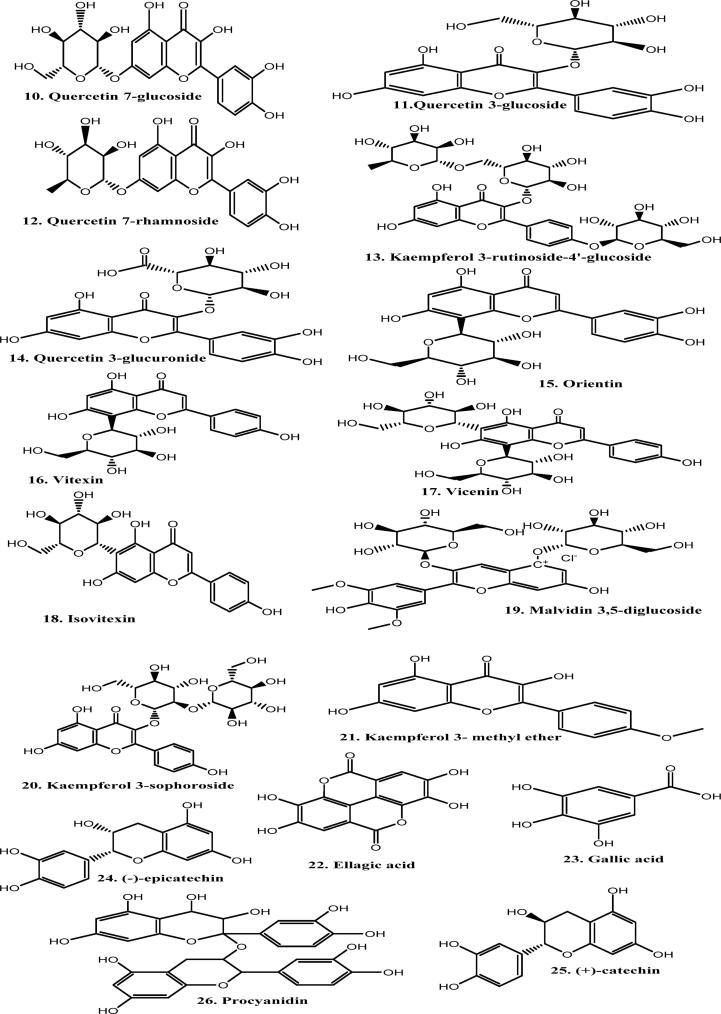
Structure of different phytochemical compounds (Leaf, floral, and exudate flavonoids; hydrolyzable and condensed tannins) identified from different species of the *Geranium* genus.

The photochemistry of about 300 temperate species of the *Geranium* genus is now generally well understood (Mabberley, 1997). Hegnauer’s chemistry of dictionary states that a minimum of 55 species have undergone chemical analysis (Hegnauer, 1966). Additionally, extensive research has been done on three familiar species, including *G. robertianum* (commonly known as Herb Robert, a European medicinal plant), *G. macrorrhizum* (an oil-obtaining plant), and *G. thumbergii* (a Japanese medicinal species). The ellagitannin geraniin, which received its name after crystallizing from *Geranium thunbergii*, it is the *Geranium* genus's most unique single compound. This secondary metabolite is found in all types of leaves in the *Geranium* species. Secondary metabolites of plants are extracted mainly by Cold maceration and Soxhlet extraction method, as shown in Fig. 1. When *G. thunbergii* leaf extracts are taken orally, geraniin (1), in contrast to nearly all other hydrolyzable tannins, does not produce an astringent flavour (Okuda, Yoshida, & Hatano, 1992). *Geranium* species are well recognized for being rabbit-proof, and the high level of leaf tannin is thought to be the cause of this potential*.* Germacrone is another naturally occurring compound whose name likely derives from the *Geranium* genus; large quantities of the sesquiterpene compound germacrone were obtained from the *G. macrorrhizum*. Geraniol (a monoterpene) (2) is also obtained from the *G. macrorrhizum*, derived from geraniol oil obtained from *Pelargonium*, a closely related genus of *Geranium. Geranium* oil cannot be considered a feature of *Pelargonium* or *Geranium* because it is found in the oils of hundreds of medicinal plants across nature. There is very little data on germacrone or geraniol extensively found in *Geranium*. Most research has focused on the flavonoid components, namely two classes of phenolic tannins (Bhat et al., 2023) . The whole diversity of plant polyphenols found in the aerial and root portions of *Geranium* is described in the current account. The structure of different phytochemical compounds identified from different species of the genus *Geranium* is shown in Fig. 2.

### 3.1 Essential oils

*G. macrorrhizum* aerial portions produced an essential oil including two familiar monoterpenoids i, e β-citronellol and geraniol (2), in addition to many sesquiterpenes. These include α-elemene, α-curcumene, and germacrone (3), which together account for up to 50 % of the oils (I. Ognyanov et al., 1958). The essential oils of other species of *Geranium* are not well documented.

### 3.2 Leaf flavonoids

Leaf flavonoids are mainly flavonol, and widely recognized querstein (4) is prevalently present. According to (Bate-Smith, 1972), quercetin is usually accomplished by the lesser homologue kaempferol (5) in 93 % of the sample and by the greater homologue myricetin in 13 % of the sample analysis of acid-hydrolyzed leaf tissue from 78 plant species. The geographic distribution of the species is somewhat associated with variation in this fundamental flavonol pattern. Plants from the central Eurasian region have a primitive pattern, which includes myricetin (6) as the predominant compound. In contrast, American and Mediterranean species exhibit an advanced pattern characterized by high kaempferol. Even though it is evident that the above-mentioned three flavonol compounds exist in the glycosidic combination in the *Geranium* genus, there are few studies on the glycosidic pattern among most species.

Additionally, a survey on the use of HPLC on *Geranium* leaves was conducted by (Okuda et al., 1980). These researchers have revealed that the 3-galactoside known as hyperin (7) frequently occurs in the genus as quercetin (9), which was observed in ethanolic extracts of 12 out of 15 *Geranium* species studied. The concentration of hyperin ranges from 0.03 to 1.6 % dry weight, with an average range of 0.43 %. In *G. thunbergii* (Japanese species), quercetin 3-galactoside (8) appears to be deficient; however, leaves of this plant consist of either combination of kaempferol 3,7-dirhamnoside (9) and kaempferol 3-arabinoside-7-rhamnoside or separately kaempferol 3-rhamnoside (10) (Harborne & Williams, 2002).

The flavonol glycosides of the medicinal plant *G. robertianum*, have been well studied; six monoglucosides are obtained from aerial parts, which include quercetin 7-glucoside (11), quercetin 3-galactoside (8), kaempferol, quercetin 3-glucoside (12) and quercetin 7-rhamnoside (13). Along with monoglucosides, there are seven 3-diglycosides, and only four out of seven were characterized entirely, including 3-rutinosides and 3-rhamnosylgalactosides of quercetin and kaempferol. Although it is unclear if *G. robertianum* varies in the amount of flavonol glycosides, it should be noted that (Okuda, Mori, & Hatano, 1980) were unable to identify the quercetin 3-galactoside mentioned by (Kartnig & Bucar-Stachel, 1991) in their specific sample.

Five native Egyptian *Geranium* species have been characterized in diverse ways by four additional flavonol glycosides that have not yet been studied. Kaempferol 3-rutinoside-4′-glucoside (14) is the most distinctive, observed in *G. rotundifolium* and *G. yemense*. In *G. dissectum,* quercetin 3-glucuronide (15) is present remarkably (Saleh, El-karemy, Mansour, & Fayed, 1983). In the above-mentioned five Egyptian species, quercetin 3-galactoside is lacking. According to (Harborne & Williams, 2002), the *Geranium* genus also contains other flavonoids, such as glycosylflavones; However, they have not been studied further. They have been observed mainly in the case of *G. phaeum*. Compared to five glycosyl flavones: orientin (16), vitexin (17), vicenin (18), and isovitexin (19), flavonol glycosides derived from quercetin are minor components (Boutard & Lebreton, 1975).

### 3.3 Floral flavonoids

The majority of *Geranium* species feature attractive blooms that range in colour from purple, blue, and red to white and pink. Those floral colours are primarily due to anthocyanins and flavonol glycosides, but very little investigation has been done on anthocyanins in the genus *Geranium*. An extensive investigation has been carried out in bluish-purple flowers in *G. sanguinea* and *G. pratense* and the Johnsons blue, a cultivated hybrid obtained from *G. pratense* and *G. himalayense*. The three plants listed above contain the main anthocyanin malvidin 3,5-diglucoside (20) (Markham, Mitchell, & Boase, 1997). Petals of these *Geranium* species contain four other flavonol glycosides in addition to anthocyanin, which include; 3-sophorosides of kaempferol, 3-glucosides, and 3-sophorosides of myricetin. Invitro studies have revealed that 3-sophorosides of kaempferol (21) are the essential co-pigment that imparts a blue colour to the flowers of plants. Moreover, a cell sap pH of between 6.8 and 6.6 is responsible for the distinctive features of floral colour development in *Geranium* species. Although it appears to be a singular property of nature, this is essential to the full-colour intensity seen in these blossoms (Markham et al., 1997).

### 3.4 Exudate flavonoids

Trichomes or glandular hairs are frequently present on the upper leaf surface of *Geranium* species. By gently rinsing leaf surfaces in a solvent like acetone, it is possible to study the chemical components of these trichomes independently of the components found inside leaves. In addition to the hydrocarbons and terpenoids usually found at the surface, the species leaves consist of a combination of lipid-soluble flavonoids, typically flavonol methyl ethers. These phytocompounds have been discovered from the leaves of *G. lucidum* and *G. macrorrhizum*. These two species of plants consist of some myricetin, quercetin, or 14 kaempferol methyl ethers. (Ivancheva & Wollenweber, 1989; I. V. Ognyanov, 1972) revealed that the leaf surface of *G. macrorrhizum* consists of kaempferol 3- methyl ether (22) and 3,5,7.2′,4,6′- hexahydroxyflavone (a novel flavonol). The evidence supporting the new flavonol has been reexamined, and it appears that a known flavonol was mistakenly identified.

### 3.5 Hydrolyzable tannins

The chemical geraniin, which was initially crystallized from *G. thunbergii* leaf extracts, is the main hydrolyzable tannin of the genus *Geranium*. Japan has traditionally utilised this plant for medicinal purposes. Over the years, many people have taken aqueous extracts of *G. thunbergia* as an antidiarrheal to regulate digestive function (Okuda et al., 1992). More than 10 % of the dried leaf's weight is made up of geraniin. Interestingly, geraniin does not possess the typical astringency associated with plant tannins; instead, it crystallizes as yellow substances. Geraniin is a chemical compound derived from a glucose molecule with two hexahydroxygallic acid residues disubstituting the 2,4 and 3,6 positions. Moreover, the sugar's C-1 has a galloyl ester group attached. Gallic acid is converted synthetically into geraniin by employing pentagalloylglucose as a precursor. Since HPLC has found geraniin in each of the 15 species examined, it would seem that geraniin is the distinctive hydrolysable tannin of the genus *Geranium* (Okuda et al., 1980).

Geraniin and its related molecule, ellagic acid (23), co-occur in the *Geranium* plant leaves. According to (Bate-Smith, 1962), the leaves of four out of the six plants he surveyed, *G. robertianum*, *G. meeboldii*, *G. phaeum,* and *G. sylvatixum* contained ellagic acid. The main sites for producing ellagic acid are rhizomes and roots of the plants, which have been observed in some sixty-one plant species (Hegnauer, 1966). Gallic acid (24), which is thought to be an ellagic acid precursor, has also been consistently seen in the roots (Hegnauer, 1966) and leaves (Bate-Smith, 1962). Gallic acid (phenolic acid) has been identified from the roots of *G. nepalensis*, *G. pratense*, and *G. maculatum*.

### 3.6 Condensed tannins

Both hydrolyzable and condensed tannins are present in the *Geranium* species, although their distribution in various organs varies greatly. According to (Bate-Smith, 1972), the rootstock is the main site where proanthocyanidin (condensed tannin) is mainly found. Only a few of the sixty *Geranium* species studied had considerable proanthocyanidin content in their leaves. These species are *G. incanum* (South Africa), *G. platypetalum* (Armenia), *G. sinense* (China), *G. lindenianum* (Venezuela), *G. renardii* (Caucasus), and *G. polyanthes* (Eurasia). *Geranium* contains proanthocyanidins based on prodelphinidin, procyanidin, or a combination of the two. However, the two procyanidin precursors, (−)-epicatechin (27) and (+)-catechin (26), have been found in the roots of *G. palustre* and *G. pratense*, suggesting that the procyanidins are most likely of a common kind of proanthocyanidins (Hegnauer, 1966). It has been determined that the levels of ellagitannin and procyanidin (25) in fresh rhizomes of *G. sylvaticum* are roughly equal. In contrast, *G. pratense* has just one-seventh of the procyanidin content and six-sevenths of the ellagitannin content (Hegnauer, 1966). *Geranium* species have historically been used as significant sources of tanning material in the leather industry due to the high tannin amount in their roots. This conventional method has been employed with at least two species; *G. wallichianum* and *G. nepalense*.

### 3.7 Miscellaneous constituents

In *G. viscosissimum* and *G. richardsonii*, tartaric acid is typically accumulated in the aerial portions. In the members of the Geraniaceae family, this organic acid is frequently present; however, this organic acid is not always found in all *Geranium* species (Stafford, 1961). When *G. sanguineum* and *G. robertianum* were analyzed, tartaric acid was absent, but citric and malic acids were present (Harborne & Williams, 2002).

## 4 Medicinal uses of *Geranium* species

### 4.1 Ethnomedicinal importance

Since ancient times, the species of *Geranium* have been utilized in numerous conventional health systems across the globe, such as Indian Ayurveda, Traditional Chinese medicine, and various indigenous medical practices, by means of herbal formulations (Williamson, 2002b). Accordingly, it has been claimed that many plants in this genus are utilised to cure a variety of illnesses, as shown in Table 1.

**Table 1:** Ethnomedicinal importance of various species of the *Geranium* genus, including *G. pratense, G. robertianum, G. wallichianum, etc*. The species of this genus are mainly found in China and India.

Species of the *Geranium* Genus	Country name	Ethnomedicinal importance	References
*G. pratense*	Europe, China, and Japan	Bacillary dysentery (Acute)	(Williamson, 2002b)
*G. robertianum*	USA, Japan, Europe, North Africa, China, South America, and India	Hemorrhage, dispersal of kidney, diarrhoea, mouthwash, wounds, gall stones, and burns.	(Williamson, 2002b)
*G. wallichianum*	India	Toothache, headache, otorrhoea, rheumatic pain, diarrhoea, body pain, backache, fever, astringent, ophthalmia, cough and cold, styptic, cough, wounds, dysentery, and jaundice	(Agnihotri et al., 2014, Kumar et al., 2015, Shaheen et al., 2012, Thakur et al., 2016)
*G. aculeolatum*	Burundi	Diarrhoea, ringworm, and purulent rashes	(Ngezahayo, Havyarimana, Hari, Stévigny, & Duez, 2015)
*G. ayavacense*	Peru	Astringent, gingivitis, hypoglycaemic, gastric lesions, ulcerative stomatitis, and gastritis.	(Aranda-Ventura, Villacrés, Mego, & Delgado, 2014)
*G. himalayense*	India	Indigestion	(Agnihotri et al., 2014)
*G. macrorrhizum*	Poland, Bulgaria, and Romania	Dysentery, antiviral, diarrhoea, gastrointestinal ulcers, styptic in haematuria, and menorrhagia	(Williamson, 2002b)
*G. lucidum*	India	Astringent and Diuretic	(Agnihotri et al., 2014)
*G. dissectum*	Lebanon	Rheumatic disorders	(Marc, 2008)
*G. maximowiczii*	China	Rheumatic disorders	(Küpeli, Tatli, Akdemir, & Yesilada, 2007)
*G. molle*	Portugal	Cancer, stomach ache, uterus inflammation, gingivitis, antiseptic and eye inflammation	(Neves, Matos, Moutinho, Queiroz, & Gomes, 2009)
*G. nepalense*	India	Astringent, ulcers, jaundice, endometriosis, itching, antibacterial, stomach disorders, renal disorders, fever, wounds, diuretic, toothache, eczema, and diarrhoea	(Agnihotri et al., 2014, Dutt et al., 2015, Singh and Rawat, 2011, Williamson, 2002b)
*G. rivulare*	India	Ulcers and Insect bites	(Williamson, 2002b)
*G. polyanthes*	India	Headache and ulcers	(Singh & Rawat, 2011)
*G. platyanthum*	China and Japan	Pain, numbness of limbs, and rheumatic disorders	(Williamson, 2002b)
*G. pusillum*	India	Wounds, analgesic, and astringent	(Agnihotri et al., 2014)
*G. purpureum*	Portugal	Cancer, hepatic protective, gall-bladder ailments, Antiulcerative, gastritis, intestinal antispasmodic, vulnerary, sea-sickness, analgesic, and gastric protective	(Novais et al., 2004, Singh and Rawat, 2011)
*G. ruizii*	Peru	Diabetes, inflammation, and chronic diarrhea	(Ikeda et al., 2014)
*G. seemannii*	Central America, Mexico, and the Caribbean	Obesity, laxative, and diuretics	(Alonso-Castro, Domínguez, Zapata-Morales, & Carranza-Álvarez, 2015)
*G. sibiricum*	India	Wounds, astringent, and diuretic	(Agnihotri et al., 2014)
*G. niveum*	Mexico	Analgesic, purgative, infectious diarrhoea, gastrointestinal disorders, fever, kidney pain, urological problems, diabetes, skin tumours, dermatological conditions	(Alonso-Castro et al., 2011, Calzada et al., 1998)
*G. phaeum*	Bulgaria, Serbia	Astringent, inflammation of gastric mucous membranes, aphrodisiac	(Chalchat, Petrovic, Maksimovic, & Gorunovic, 2002)
*G. mexicanum*	Mexico, Venezuela	Laxative in infants, antispasmodic, rashes, wounds	(Williamson, 2002a)
*G. incanum*	South Africa	Diarrhoea, menstruation	(Amabeoku, 2009, Steenkamp, 2003)
*G. bellum*	Mexico	Fever, pain, gastrointestinal disorders	(Bautista et al., 2015)
*G. carolinianum*	China	Diarrhoea, rheumatic arthritis	(Li et al., 2008)
*G. core-core*	Chile	Cataracts, shock, fever, astringent, toothache, inflammatory conditions	(Rodriguez et al., 1994)
*G. koreanum*	China	Itching, bruising, enteritis, chronic diarrhoea, liver disorders	(Oh et al., 2015)
*G. strictipes*	China	Enteritis, diarrhoea, chronic gastritis	(Zuo et al., 2008)
*G. tuberosum*	Cyprus	Cardiovascular, skin	(González-Tejero et al., 2008)
*G. wilfordii*	China	Chronic rheumatism, gastrointestinal disorders, diarrhoea, dysentery	(Williamson, 2002a, Zhang et al., 2013)
*G. sanguineum*	Eastern Europe	Haemorrhage, diarrhoea	(Williamson, 2002a)

### 4.2 Pharmacological importance

In parallel with the growing fascination with herbal remedies overall, a comprehensive assessment of the characteristics of medicinal plants belonging to the genus *Geranium* has been undertaken, mainly over the previous 20 years. Many studies examining various biological features of a reasonably broad number of species from this genus can currently be found in the specialized literature.

#### 4.2.1 Antibacterial activity

**Fig. 3:**
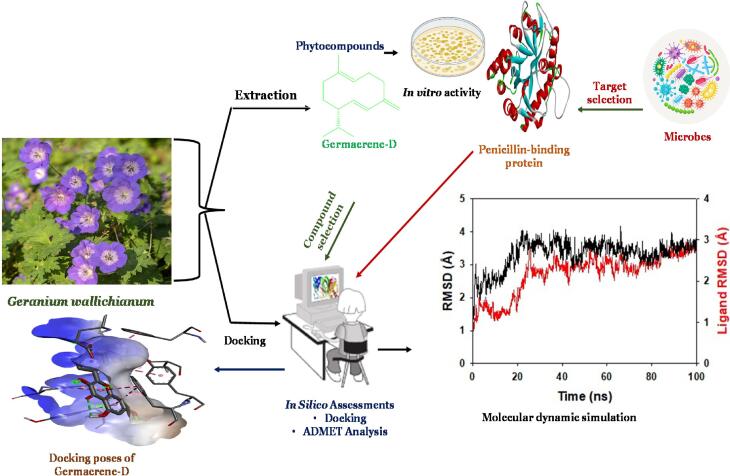
The general overview of antimicrobial activity of the *Geranium* genus. Phytocompounds were identified using the LC-MS technique, followed by *in-silco* molecular docking and MD simulation.

Many researchers have studied the antibacterial properties of the *Geranium* genus. The general overview of the antimicrobial activity of the *Geranium* genus is shown in Fig. 3. The extracts of many species of *Geranium* were tested against various bacterial strains, as shown in Table 2. This investigation primarily used aqueous and alcoholic extracts of plants of different geographic origins. The two main screening methods used to evaluate the antibacterial potential were broth microdilution and disc diffusion methods. A broad range of inhibitory action was detected in the extracts of plants, and in most cases, minimum inhibitory concentration (MICs) was reported. The antimicrobial potential of *Geranium* species (essential oils) has been far less studied than that of solid–liquid extracts. Several hydrodistilled essential oils were tested for their ability to inhibit various bacterial strains, including several plant pathogens. Research studies have evaluated the antimicrobial potential of the *Geranium wallichianum* dry extracts (ethanolic, ethyl acetate, methanolic and petroleum ether), as shown in Table 3 (Mir et al., 2022). Three fungal strains that had their antifungal potential assessed were among the nine microbial strains identified for the investigation, whereas six were bacterial strains such as *N. mucosa, K. pneumoniae, M. luteus, S. pneumonia, E. coli, H. influenzae, C. paropsilosis C. albicans*, and *C. glabrata* were chosen. The antimicrobial potential of different *G. wallichianum* extracts (ethanolic, petroleum ether, ethyl acetate, and methanolic) was obtained in this study. The antibacterial efficacy of various *G. wallichianum* extracts has exhibited remarkable antimicrobial potential against diverse microbial isolates. MICs of positive antifungal and antibacterial drugs like amphotericin B and ciprofloxacin, respectively, were determined through the broth dilution method. The MIC of various extracts demonstrated significant antimicrobial properties. The ethyl acetate extracts showed the highest antimicrobial potential compared to the other three extracts. The MIC values of the *G. wallichianum* extract (ethyl acetate) against *H. influenzae, M. luteus, K. pneumoniae, E. coli, N. mucosa and S. pneumoniae* were 6.25, 3.12, 25, 100, 25 and 12.5 μg/mL, respectively*.* Compared to various bacterial strains, plant extracts demonstrated less efficient antimicrobial potential against the three fungal strains, such as *C. paropsilosis*, *C. glabrata*, and *C. albicans*.

**Table 2:** MIC (Minimum inhibitory concentration) values of various *Geranium* species essential oils against different bacterial strains. Various positive controls were taken for the determination of MIC: Ampicillin, Streptomycin, Chloramphenicol and Tetracycline.

*Geranium* species	Part used	*Bacillus cereus*	*Bacillus cereus*	*Bacillus subtilis*	*Micrococcus flavus*	*Mycobacterium smegmatis*	*Staphylococcus aureus*	*Staphylococcus aureus*	*Streptococcus agalactiae*	*Staphylococcus pseudintermedius*	*Streptococcus canis*	*Staphylococcus aureus*	*Clostridium perfringens*	*Listeria monocytogenes*	References
***G. asphodeloide***	**aerial portion**	**^–^**	**0.355**	**–**	**–**	**0.355**	**–**	**–**	**–**	**–**	**–**	**3.50**	**^–^**	**–**	(Uzun et al., 2004)
***G. columbinum***	**aerial parts**	**–**	**–**	**14**	**7**	**–**	**1.750**	**–**	**–**	**–**	**–**	**6**	**0.437**	**–**	(Radulovic et al., 2011)
***G. lucidum***	**whole part**	**–**	**–**	**13.4**	**13.4**	**–**	**3.35**	**–**	**–**	**–**	**–**	**0.312**	**1.675**	**–**	(Radulovic et al., 2011)
***G. macrorrhizum***	**aerial portion**	**–**	**–**	**0.001**	**–**	**–**	**0.039**	**–**	**–**	**–**	**–**	**0.625**	**–**	**–**	(Radulović et al., 2010)
***G. psilostemon***	**aerial portion**	**–**	**–**	**–**	**–**	**4.220**	**–**	**–**	**–**	**–**	**–**	**–**	**–**	**–**	(Renda et al., 2016)
***G. purpureum***	**aerial portion**	**–**	**4.22**	**–**	**–**	**3.365**	**–**	**–**	**–**	**–**	**–**	**–**	**–**	**–**	(Renda et al., 2016)
***G. pyrenaicum***	**aerial portion**	**–**	**3.365**	**–**	**–**	**0.335**	**0.335**	**–**	**–**	**–**	**–**	**–**	**–**	**–**	(Renda et al., 2016)
***G. robertianum***	**leaves**	**–**	**0.16**	**–**	**–**	**–**		**1.25**	**5**	**2.5**	**2.5**	**–**	**–**	**–**	(Renda et al., 2016)
***G. sanguineum***	**whole plant**	**5**	**–**	**–**	**–**	**–**	**5**	**–**	**–**		**–**	**5**	**–**	**–**	(Renda et al., 2016)

**Table 3:** *In-vitro* antimicrobial potential of different extracts of *Geranium wallichianum* D. Don using Broth dilution method. Positive antimicrobial drugs were also taken.

Strains	MIC (µg/mL)				
	ETH	MT	PE	EA	AMF-B/CIP
***K. pneumoniae***	25	6.25	25	25	0.039
***E. coli***	50	100	100	100	0.625
***N. mucosa***	25	25	25	25	03.12
***S. pneumoniae***	25	25	12.5	12.5	0.625
***M. luteus***	3.12	6.25	1.56	3.12	1.25
***H. influenzae***	25	25	25	6.25	1.25
***C. glabrata***	400	400	400	400	2 0.5
***C. Paropsilosis***	400	400	400	400	2 0.5
***C. albicans***	6.25	200	400	400	1.25

Where CIP; Ciprofloxacin (standard antimicrobial drug and AMF-B: Amphotericin-B (standard antifungal drug); PE: petroleum ether, ETH; ethanolic, MT; methanolic, and EA: ethyl acetate.

#### 4.2.2 Anticancer activity

Kosuge et al. (1985) first examined the anticancer potential of the plants of the *Geranium* genus; they carried out this work on *G. nepalense* (Kosuge et al., 1985). In this study, 90 Chinese herbal species were supposed to have anticancer potential; the *G. nepalense* extracts (methanolic and aqueous) were among the few to demonstrate considerable *invitro* cytotoxic effects against HeLa (cervical cancer cells). At a 0.1 mg/mL dosage, both extracts showed more than 75 % growth inhibition.

Kashiwada et al. (1992) revealed that aqueous acetate (80 %) extract from *G. thunbergii* has an effective cytotoxic effect against RPMI-7951 melanoma cancer cells with an ED_50_ value of 20 µg/mL (Kashiwada, Nonaka, Nishioka, Chang, & Lee, 1992). Nearly 900 natural product extracts in comparison to paclitaxel were subjected to high-throughput screening for their ability to suppress the division of MDA-MB-231 cells by having an antimitotic impact. *G. maculatum* (ethanolic extract) exhibits a moderate growth inhibition potential (IG_50_ value of 0.06 mg/mL) (; E. Mazzio, Badisa, Mack, Deiab, & Soliman, 2014). According to studies by Mazzio and Soliman, the *G. maculatum* extract (ethanolic) was also found to be cytotoxic to the Neuro 2-a murine neuroblastoma cell line, with an LC_50_ value of 1.170 mg/ml (E. A. Mazzio & Soliman, 2009). Kim (2016) observed that aqueous ethanolic extract (70 %) of *G. krameri* possesses low cytotoxicity against a B16F10 murine skin cancer cell line (ID_50_ value of 469 µg/mL) (H.-S. Kim, 2016).

Different aqueous (infusion and decoction) and organic extracts (ethyl acetate, acetone, n-hexane, and methyl chloride, obtained by successive extraction from *Geranium robertianum* were assessed against various human cancer cell lines, such as cervical (HeLa), non-small cell lung (NCIH460), breast (MCF-7) and hepatocellular (HepG2) hepatocellular (HepG2) carcinomas (Mir et al., 2023;Barros et al.). All these extracts possessed cytotoxic potential (GI_50_ values range of 55.68–236 µg/mL). The acetone extract of *G. robertianum* has the most significant cytotoxic effect with GI_50_ values from 57 to 60 µg/mL against various human cancer cell lines. Ellipticine, a potent anti-cancer drug, was utilized as a positive control and showed GI50 ranges between 0.91 and 2.29 µg/mL. The same author conducted a similar investigation on *Geranium molle* against the similar above-mentioned cell lines, which revealed the same findings with the extract of acetone, showing GI_50_ values ranging from 50 to 85 µg/mL (Graça et al., 2016b).

Sohretoglu et al. examined the cytotoxic potential of various *G. tuberosum* and *G. psilostemon* extracts; often employed against a KB human epidermoid cancer cell line in traditional Turkish medicine (Şöhretoğlu, Genç, & Harput, 2017). Various extracts of these two plants, such as aqueous, 80 % ethyl acetate, methanol, petroleum ether, and butanol, showed a concentration-dependent cytotoxic effect in the range of 10–0.1 µg/mL. *G. tuberosum* and *G. psilostemon* aqueous extracts showed proliferation inhibition of approximately 65 % and 55 %, respectively, when compared to doxorubicin (standard) at a concentration of 10 µg/mL. The butanol and ethyl acetate extracts exhibited less than 30 % anticancer potential at the same concentration.

**Fig. 4:**
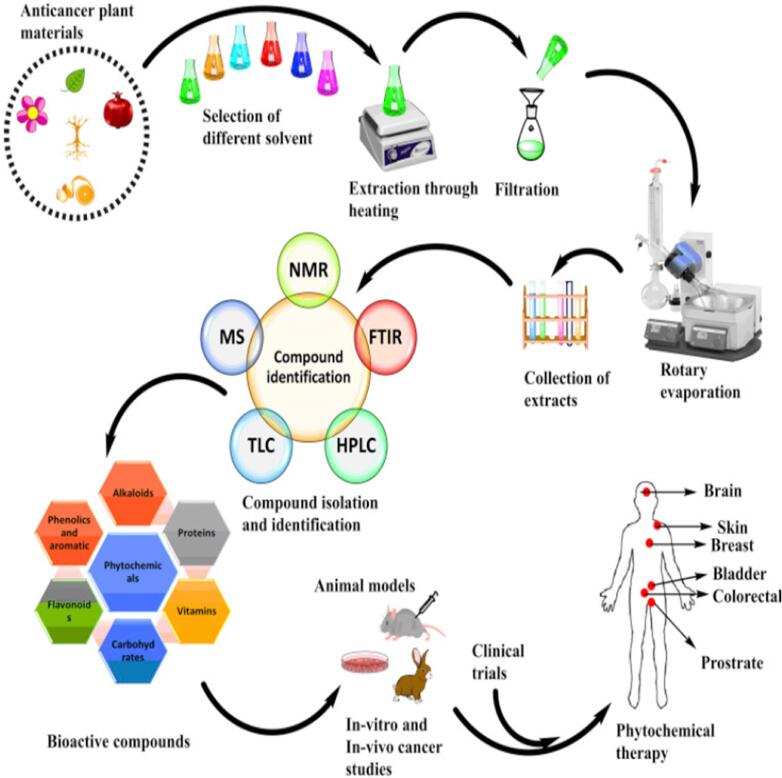
The general view of anticancer activity of plant extracts of the *Geranium* genus. After the extraction of plant material, compounds were identified using TLC, HPLC and NMR techniques. These compounds were subjected to *in-vitro* and *in-vivo* anticancer studies.

Herrera-Calderon et al. (2018) recently evaluated the cytotoxicity of *G. ruizii* aqueous ethanol extract against different breast cancer cell lines; H-460, MCF-7, HT-29, K-562 (myelogenous leukaemia), M-14 (melanoma) and Du-145 (prostate) (Herrera‑Calderon et al., 2018). The extract DU-145 cell line had strong anticancer potential with an IC_50_ value of >15.63 µg/mL. The anticancer potential of positive control standard; 5-fluorouracil is more than plant extracts with IC_50_ values ranged from 0.33 to 4.08 µg/mL. The general view of the anticancer potential of plant extracts is shown in Fig. 4.

#### 4.2.3 Antidiarrhoeal potential

Different *Geranium* species are used internally to treat diarrhoea problems (Williamson, 2002b, Williamson, 2002a), but many *Geranium* species have yet to be explored. Secretory diarrhea, which develops when water is secreted into the intestinal lumen rather than absorbed, is one of the mechanisms in diarrhea etiology and can quickly result in severe dehydration (Field, 2003). In Sprague-Dawley rats, extracts of *G. mexicanum* (aerial portions) were reported to exhibit antisecretory action (Velázquez, Calzada, Torres, González, & Ceballos, 2006). The aqueous plant extract demonstrated 42.1 per cent inhibition compared to loperamide (43.3 %); it is taken as a standard for the treatment of diarrhoea. However, the methanolic extract exhibits a more significant antisecretory potential of 93.4 % inhibition than positive control loperamide. Both methanolic and aqueous extracts of the roots lack antisecretory potential.

Amabeoku (2009) revealed that the aqueous extract (*G. incanum* leaves) had antipropulsive and antidiarrhoeal potential in albino mice with oil-induced diarrhoea, lowering various diarrhoeal episodes and decreasing the faecal output, with a net outcome same as that of loperamide (Amabeoku, 2009). In Wistar rats, diarrhoea induced by castor oil, *G. ocellatum* aqueous leaf extract, showed a remarkable anti-diarrheal activity, significantly lowering the total amount and weight of wet faeces. A maximum of 78.87 % was observed when diarrhoea rats were treated with the plant extract, and 79.52 % inhibition was observed when treated with loperamide (George & Joseph, 2012).

#### 4.2.4 Anthelmintic potential

Acharya et al. (2014) revealed that the methanolic extract of *G. viscosissimum* (leaves) at a dosage of 50 mg/ml in Dimethyl sulfoxide could prevent invitro egg hatch of *Haemonchus contortus* with EG_50_ of 0.63 mg/mL (Acharya, Hildreth, & Reese, 2014). *Haemonchus contortus* is a gastrointestinal nematode parasite that severely reduces livestock production (Preston et al., 2014). *G. incanum* methanolic extract collected after the sequential plant extraction method with methylene chloride, ethyl acetate, and n-hexane was observed to induce approximately 85 per cent larvae paralysis of *Haemonchus contortus* (20 mg/ml) within 24 h of contact (Olalekan, Robert, & Thozamile, 2015).

#### 4.2.5 Anti-inflammatory potential

Kupeli et al. (2007) assessed the anti-inflammatory potential of *G. finitimum* aqueous extracts, which was produced by partitioning a crude methanolic extract between water and chloroform, using three inflammation models; Swiss albino mice, prostaglandin E2, and carrageenan-induced paw oedema and TPA induced ear oedema (Kupeli, 2007). Positive control anti-inflammatory drug (indomethacin) with a concentration of 10 mg/Kg, the plant extract at the concentration of 100 mg/Kg, significantly reduced both carrageenan (26.6 % inhibition after 3 hrs post-injection, compared to 38 per cent for indomethacin), and prostaglandin E2 induced paw oedema (25.3 % inhibition after 24 min post-injection, compared to 13 per cent for indomethacin) also TPA induced ear oedema weight (42.4 % inhibition after 4 hrs of post-injection, compared to 59.7 % for indomethacin). An intragastrically administered dose of 1.69 g/Kg of a 50 % aqueous-ethanol *G. wilfordii* extract significantly reduced the thickening in the paw oedema caused by carrageenan one hour after carrageenan injection in Sprague-Dawley rats for five days. This effect was much stronger than the 0.1 mg/kg of acetylsalicylic acid, which is taken as a positive control (Huang et al., 2015). The extract demonstrated potential action against the TNF-α, an essential signalling protein in many inflammatory responses, under the influence of concentration, according to research done in vitro by the same authors using L929 murine fibrosarcoma cells. Anti-inflammatory potential in percentage inhibition of various extracts of *Geranium* species such as methanol, ethanol, ethyl acetate, and Aqueous using different *in vivo* animal models is shown in Table 4.

**Table 4:** Anti-inflammatory potential in percentage inhibition of various extracts of *Geranium* species such as methanol, ethanol, ethyl acetate, and Aqueous using different *in vivo* animal models. Positive controls (standard) are also used, such as indomethacin, aspirin, and diclofenac.

*Geranium* species	Extract used/standard	Methods/Animal Models	Conc. of extract	Conc. of standard	%age Inhibition of extract	%age Inhibition of standard	References
*G. finitimum*	Methanol/Indomethacin	Carrageenan-induced paw oedema	100 mg/Kg	10 mg/Kg	26.6	38 %	(Küpeli et al., 2007)
*G. wilfordii*	Ethanol/Aspirin	Carrageenan-induced paw oedema	1.69 g/kg	0.1 mg/kg	33.3 %	35.6	(Huang et al., 2015)
*G. thunbergii*	Ethanol	LPS stimulated RAW 264.7 cells	50 µg/mL	–	60 %	–	(Sung et al., 2018)
*G. bellum*	Aqueous acetone/diclofenac	carrageenan-induced paw oedema	300 mg/kg	30 mg/Kg	41.1 %	47.2 %	(Velázquez-González et al., 2014)
*G. sibiricum*	Ethanol	Phorbol-12-myristate 13-acetate plus calcium ionophore A23187 (PMACI)	50–200 mg/mL	–	52 %	–	(Shim, Oh, & Lim, 2009)
*G. nepalense*	Ethyl acetate/aspirin	TPA-induced ear oedema	2.5 g/Kg	0.6 g/kg	–	–	(Lu, Li, Li, Liang, & Shen, 2012)
*G. pratense*	Aqueous/indomethacin	PGE2-induced hind paw edema	(100 mg/kg	10 mg/kg	38.4 %	32.4 %	(Piwowarski et al., 2014)
*G. carolinianum*	Aqueous/indomethacin	Fresh egg white-induced acute paw oedema	500 mg/Kg	5 mg/Kg	40.5 %	69.7	(Li et al., 2016)
*G. koreanum*	Dichloromethane	Acute Reflux Esophagitis-Induced Rats	200 µg/mL	–	89 %	–	(Nam, Nan, & Choo, 2018)

#### 4.2.6 Antioxidant potential

**Fig. 5:**
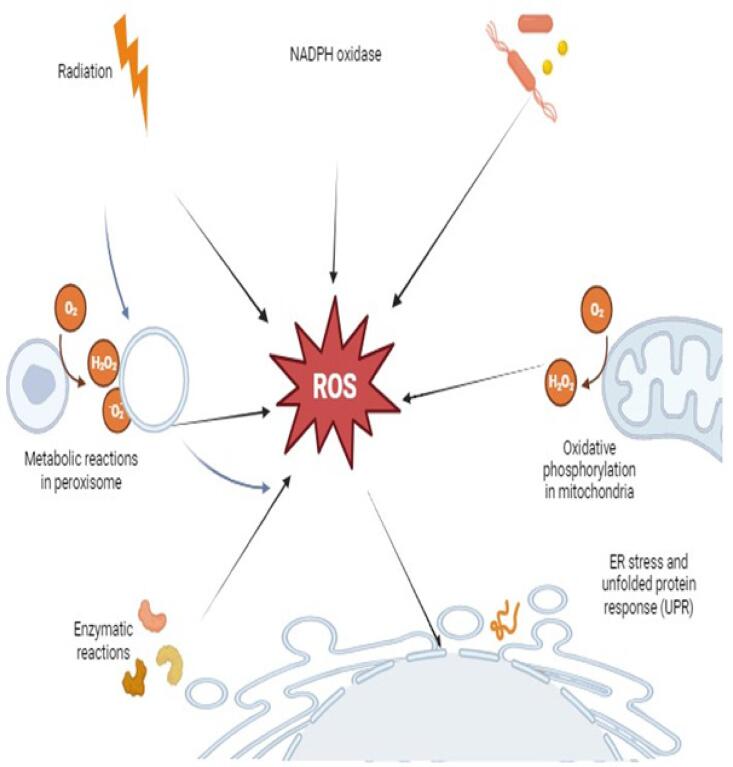
Generation of radical oxygen species (ROS) in the cell in different ways, including radiations, cell uptake of microbes, enzymatic reaction, etc.

Reactive oxygen species (ROS) consists of alkoxyl, hydroperoxyl, hydroxyl, superoxide, and alkoxyl radicals (Mehraj et al., 2022). Two nitrogen free radicals include nitric acid and nitrogen dioxide. Free radicals of nitrogen and oxygen can be transformed into reactive species that are not radicals, like hypochlorous acid, hydrogen peroxide, and peroxynitrite. Under both pathological and physiological circumstances, aerobic cells produce reactive nitrogen species, ROS, and reactive chlorine species (Evans & Halliwell, 2001). Thus, radical and non-radical species are included in RNS and ROS. The antioxidant system keeps these species at extremely low steady-state concentrations, but when their formation rises, they could be able to outpace the antioxidant system’s scavenger function, causing oxidative stress and harm to biological targets **(**Fig. 5**)**. The antioxidant potential has been the most evaluated biological activity in the *Geranium* genus; there are about 30 species of *Geranium* studied in various geographical regions. Different analytical methods have been utilized to assess the antioxidant potential of various *Geranium* extracts. The 2,2-diphenyl-1-picrylhydrazyl (DPPH) free radical scavenging method has been used for most evaluations; it is widely utilized in vitro to determine antioxidant potential due to its speed, simplicity, and low cost in contrast to other methods (Alam & Bristi, 2013). Ferric-reducing antioxidant power (FRAP), hydroxyl radical scavenging, and reducing power are some other electron transfer-based assays that have also been utilized in some cases. The antioxidant potential of various *Geranium* species has been evaluated: *G. bellum* (Camacho-Luis et al., 2008), *G. sibiricum* and *G. robertianum* (Ben Jemia, Aidi Wannes, Ouchikh, Bruno, & Kchouk, 2013), and *Geranium purpureum* (Şöhretoğlu, Sakar, Sabuncuoğlu, Özgüneş, & Sterner, 2011). Antioxidant potential and total phenolics content of *Geranium* species were determined using various methods such as DPPH, FRAP, and ABTS, as shown in Table 5.

**Table 5:** Antioxidant potential and total phenolics content of *Geranium* species, using various methods such as DPPH, FRAP, and ABTS method. Different solvents, like ethanol, methanol, ethyl acetate, and water, were used.

*Geranium* species	Part used	Extract	Method	Antioxidant activity	Total phenolics content	References
*G. sibiricum*	Whole plant	Aqueous	DPPH (IC_50_)	2.92 µg/mL	169.46 mg GAE/g	(Wu et al., 2010)
*G. tuberosum*	Aerial parts	Ethyl acetate	DPPH (%age inhibition)	90	–	(Söhretoglu, Sakar, Ekizoglu, & Özalp, 2007)
*G. sanguineum*	Aerial roots	Methanol	DPPH (IC_50_)	13.86 ± 0.84 µg/mL	34.60 % (w/w)	(Sokmen et al., 2005)
*G. thunbergii*	leaves and Stem	Methanol	IAC water-soluble substances	598.7 ± 10.9 µmol AA/g	53.3 ± 2.8 mg GAE/g	(Kim et al., 2008)
*G. sylvicatum*	Aerial parts	Methanol/Aqueous	DPPH (IC_50_)	30 µg/mL	–	(Nikolova, Tsvetkova, & Ivancheva, 2010)
*G. tuberosum*	Aerial parts	Methanol/Aqueous	H_2_O_2_– ILP (%age inhibition)	50	–	(Şöhretoğlu, Sakar, Sabuncuoğlu, Özgüneş, & Sterner, 2009)
*G. wallichianum*	Roots	Ethyl acetate	DPPH (IC_50_)	19.05 ± 0.90 µg/mL	–	(Ismail et al., 2009)
*G. wilfordii*	Whole plant	Methanol/Aqueous	FRAP	347.33 ± 7.99 µmol Fe^2+^/g	14.98 ± 0.64 mg GAE/g	(Gan et al., 2010)
*G. lucidum*	Aerial parts	Methanol/Aqueous	DPPH (IC_50_)	45 µg/mL	–	(Nikolova et al., 2010)
*G. molle*	Whole plant	Aqueous	DPPH (IC_50_)	324 ± 9 µg/mL	79 ± 1 mg GAE/g	(Graça et al., 2016b)
*G. nepalense*	Whole plant	Ethanol/Aqueous	DPPH (IC_50_)	46.3 ± 0.84 µg/mL	169.4 ± 7.84 mg GAE/g	(Sim, Jang, Lee, Jung, & Cho, 2017)
*G. niveum*	Roots	Methanol/Chloroform	DPPH (IC_50_)	7.3 µg/mL	–	(Calzada et al., 1998)
*G. pratense*	Leaves & flowers	Aqueous	DPPH (%age inhibition)	13	–	(Myagmar & Aniya, 2000)
*G. psilostemon*	Aerial parts	EtOAc	DPPH (%age inhibition)	80	–	(Söhretoglu et al., 2007)
*G. purpureum*	Leaves	Ground material	Protection factor	3.1	–	(Proestos, Boziaris, Nychas, & Komaitis, 2006)
*G. ruizii*	Whole plant	EtOH/H2O	DPPH (%age inhibition)	23.7	–	(Söhretoglu et al., 2007)
*G. robertianum*	Whole plant	Aqueous	DPPH (EC_50_)	65 ± 1 µg/mL	228 ± mg GAE/g	(Graça et al., 2016a)
*G. pyrenaicum*	Aerial parts	MeOH/H2O	DPPH (IC_50_)	13.61 µg/mL	–	(Nikolova et al., 2010)
*G. lasiopus*	Aerial parts	Ethyl acetate	DPPH (%age inhibition)	80.143	–	(Şöhretoğlu, Ekizoğlu, Özalp, & Sakar, 2008)
*G. glaberrimum*	Aerial parts	Ethyl acetate	DPPH (%age inhibition)	90	–	(Söhretoglu et al., 2007)
*G. ayavacence*	Whole plant	Aqueous	DPPH (IC_50_)	19 µg/mL	–	(Okuhama et al., 2002)
*G. bellum*	Aerial parts	Ethyl acetate	ABTS (%age inhibition)	95	–	(Camacho-Luis et al., 2008)
*G. caeruleum*	Aerial parts	Methanol/Aqueous	DPPH (IC_50_)	30 µg/mL	–	(Nikolova et al., 2010)
*G. collinum*	Aerial parts	Ethanol/Aqueous	DPPH (IC_50_)	0.027 ± 0.002 mg/mL	131.7 ± 7.86 mg GAE/g	(Sapko, Chebonenko, Utarbaeva, Amirkulova, & Tursunova, 2016)
*G. favosum*	Whole plant	Dichloromethane	DPPH (%age inhibition)	16.38	0.254 ± 0.02 mg GAE/g	(Adam et al., 2018)
*G. columbinum*	Aerial parts	Methanol/Aqueous	DPPH (IC_50_)	30 µg/mL	–	(Nikolova et al., 2010)

#### 4.2.7 Antileishmanial potential

*Leishmania* tropica is the causative agent of the neglected tropical illness leishmaniasis (Kaye & Scott, 2011). This parasite is peculiar to approximately 100 countries and has an annual incidence rate of about 1.2 million. Conventionally prescribed antileishmanial medications are frequently ineffective, toxic and extremely costly. Antimonials were once thought to be promising treatments for leishmaniasis, but because *Leishmania tropica* has become resistant to them, the medication has lost its efficacy. Therefore, the scientific community is working to create substitute treatments for it. Since then, a lot of research has been done to create Magnetic nanoparticles (MNPs) for the treatment of leishmaniasis. Various MNPs have been used for the cytotoxic evaluation against Leishmanial parasites in various *in-vitro* investigations (Hameed et al., 2019). However, the cytotoxicity activity of biogenic NiONPs against *L. tropica* has not received much attention.

**Fig. 6:**
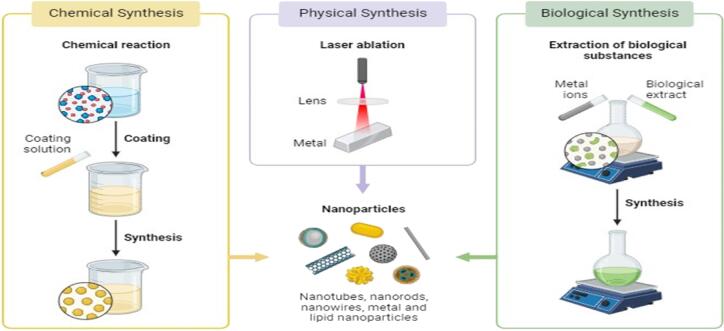
An overview of the several processes used to produce nanoparticles from plant extracts. Nanoparticles are synthesized by three methods: chemical synthesis (chemical reaction), physical synthesis (laser ablation) and biological synthesis (extraction of bioactive substances).

Research conducted by (Khalil et al., 2018) on the antileishmanial activity of biogenic NiONPs was determined against *L. tropica*. According to this study, leishmanial parasites were subjected to 1–200 μg ml^−1^ concentration of NiONPs for 72 h and demonstrated concentration-dependent suppression of *Leishmania tropica*. As the concentration of NiONPs increased, there was also an increase in antileishmanial activity. NiONPs demonstrated remarkable antileishmanial potential against *L. tropica* promastigotes with an IC_50_ value of 22.12 μg mL^−1^. Similarly, IONPs showed antileishmanial activity against *L. tropica* amastigotes with an IC_50_ of 26.58 μg mL^−1^, which is confirmed by previous investigations of biogenic NiONPs (Khalil et al., 2018). Future medications utilizing NiONP materials may have potent antileishmanial drug delivery due to reduced IC_50_ and dose dependence. An overview of various methods of synthesis of nanoparticles from plant extracts is shown in Fig. 6.

## 5 Conclusion

Many plants in the genus *Geranium* provide positive biological activities that have been scientifically proven. Many species appear to have clear therapeutic capability for a range of ailments, based on studies conducted to evaluate the corresponding biological activity. Numerous novel compounds have been shown to provide a variety of therapeutic benefits, such as anticancer activity, cholinesterase inhibition, antiparasitic activities, and antifungal and antiviral activities. These studies authenticate the wide range of the *Geranium* plant’s pharmacological applications, possibly serving as a vast drug discovery resource. Additionally, it might clarify the medicinal benefits of herbal medications derived from *Geranium*. A lot needs to be explored concerning the biological and phytochemical investigation of the phytochemistry of the *Geranium* species. The biological components of other species of *Geranium* remain unexploited. Secondly, all biological activities of isolated compounds are carried out *in-vitro* experiments, and significantly fewer reports are documented in the *in-vivo* studies. The biological activities of constituents derived from *Geranium* should be evaluated in *in-vitro* and *in-vivo* models to study and utilize this genus for diverse attributes further. The *Geranium* genus is rich in new and novel compounds, but only a few species are current study subjects. More new compounds from other species should be studied in detail in the future. The studies on the pharmacological effects of compounds derived from *Geranium* are inclusive. Still, current research is limited to extracts, so it is essential to concentrate on the effects of *Geranium* compounds and the future relationship between structure and activity.

## Funding

The research was funded by Deanship of Postgraduate Studies and Scientific Research at Majmaah University, Saudi Arabia, for supporting this work under Project Number No. R-2024-962.

## Declaration of competing interest

The authors declare that they have no known competing financial interests or personal relationships that could have appeared to influence the work reported in this paper.
